# Asymptomatic *Plasmodium falciparum* Malaria in Pregnant Women in the Chittagong Hill Districts of Bangladesh

**DOI:** 10.1371/journal.pone.0098442

**Published:** 2014-05-23

**Authors:** Wasif A. Khan, Sean R. Galagan, Chai Shwai Prue, Jacob Khyang, Sabeena Ahmed, Malathi Ram, Mohammad Shafiul Alam, M. Zahirul Haq, Jasmin Akter, Gregory Glass, Douglas E. Norris, Timothy Shields, David A. Sack, David J. Sullivan, Myaing M. Nyunt

**Affiliations:** 1 Centre for Population, Urbanization and Climate Change, International Centre for Diarrhoeal Disease Research, Bangladesh, Dhaka, Bangladesh; 2 Johns Hopkins Malaria Research Institute, Department of Molecular Microbiology and Immunology, Johns Hopkins Bloomberg School of Public Health, Baltimore, Maryland, United States of America; 3 Department of International Health, Johns Hopkins Bloomberg School of Public Health, Baltimore, Maryland, United States of America; 4 Department of Medicine, University of Maryland School of Medicine, Baltimore, Maryland, United States of America; Université Pierre et Marie Curie, France

## Abstract

**Background:**

Pregnancy is a known risk factor for malaria which is associated with increased maternal and infant mortality and morbidity in areas of moderate-high malaria transmission intensity where *Plasmodium falciparum* predominates. The nature and impact of malaria, however, is not well understood in pregnant women residing in areas of low, unstable malaria transmission where *P. falciparum* and *P. vivax* co-exist.

**Methods:**

A large longitudinal active surveillance study of malaria was conducted in the Chittagong Hill Districts of Bangladesh. Over 32 months in 2010–2013, the period prevalence of asymptomatic *P. falciparum* infections was assessed by rapid diagnostic test and blood smear and compared among men, non-pregnant women and pregnant women. A subset of samples was tested for infection by PCR. Hemoglobin was assessed. Independent risk factors for malaria infection were determined using a multivariate logistic regression model.

**Results:**

Total of 34 asymptomatic *P. falciparum* infections were detected by RDT/smear from 3,110 tests. The period prevalence of asymptomatic *P. falciparum* infection in pregnant women was 2.3%, compared to 0.5% in non-pregnant women and 0.9% in men. All RDT/smear positive samples that were tested by PCR were PCR-positive, and PCR detected additional 35 infections that were RDT/smear negative. In a multivariate logistic regression analysis, pregnant women had 5.4-fold higher odds of infection as compared to non-pregnant women. Malaria-positive pregnant women, though asymptomatic, had statistically lower hemoglobin than those without malaria or pregnancy. Asymptomatic malaria was found to be evenly distributed across space and time, in contrast to symptomatic infections which tend to cluster.

**Conclusion:**

Pregnancy is a risk factor for asymptomatic *P. falciparum* infection in the Chittagong Hill Districts of Bangladesh, and pregnancy and malaria interact to heighten the effect of each on hemoglobin. The even distribution of asymptomatic malaria, without temporal and spatial clustering, may have critical implications for malaria elimination strategies.

## Background

Pregnant women are at greater risk of acquiring malaria infection and developing symptomatic and complicated malaria disease than their non-pregnant counterparts [1]. Malaria infection during pregnancy, with or without symptoms, is a known cause of maternal anemia [1]–[4]. Massive sequestration of *Plasmodium falciparum* parasites in the placenta, with or without detectable parasites in the peripheral circulation, is a distinct feature of pregnancy-associated malaria [5], [6], and is believed to be responsible for an increased risk of adverse pregnancy outcomes including miscarriage, stillbirth, prematurity, and the delivery of a low birth-weight baby [2], [6]–[8].

The major risk factors associated with malaria during pregnancy include young maternal age, a low number of previous pregnancies (primi- or secundigravidae), and gestational age in pregnancy [9]. In addition, limited data suggest that pregnancy-associated malaria may play a critical role in pre-eclampsia [10]–[12], a serious pregnancy-associated disorder associated with negative pregnancy outcomes with worsened maternal and infant survival. In African countries with moderate to high malaria transmission, antimalarial drug combination sulfadoxine-pyrimethamine is used to protect pregnant women, regardless of malaria symptoms, from the malaria-related negative pregnancy outcomes [13]. This treatment, known as intermittent preventive treatment of malaria in pregnancy (IPTp), significantly reduces malaria-related adverse effects on the mother and the fetus and improves birth outcomes [14], [15] and has been implemented as part of routine antenatal care in 34 of 44 African countries with ongoing malaria transmission following the recommendations of World Health Organization.

Much of the knowledge about pregnancy-associated malaria is primarily based on data from sub-Saharan Africa where malaria transmission intensity is moderate to high and *P. falciparum* dominates. Data on the nature and magnitude of malaria on pregnant women and their pregnancy outcomes is relatively sparse in regions where malaria transmission intensity is low or unstable and mixed infections with *P. falciparum* and *P. vivax* are common [16]. It is generally postulated that malaria infection in such hypoendemic settings, defined as transmission in the regions where less than 10% of 2–9 year old children are positive for malaria parasites and where malaria tends to manifest itself as seasonal outbreaks, is more likely to be symptomatic compared to infections in hyperendemic settings due to lack of meaningful immunity to malaria parasites [17]. However, evidence suggests that pregnant women in hypoendemic regions may suffer from more severe and complicated disease [18], and that asymptomatic infections may be more common in the pregnant populations than expected [19].

We conducted a field clinical longitudinal study to assess whether pregnancy is a risk factor for asymptomatic *P. falciparum* malaria in the Chittagong Hill Districts (CHD) of Bangladesh. In Bangladesh, 26.9 million people residing in 13 of its 64 districts are at risk of malaria and approximately 50,000 clinical malaria cases and 100–500 malaria-related deaths were reported annually (unpublished data, Bangladesh Ministry of Health and Family Welfare, 2009). The highest incidence of malaria is found in the CHD [20], [21], a remote, forested ecozone located in Bangladesh's southeastern region contiguous with Myanmar that is primarily inhibited by 12 non-Bengali ethnic tribal groups [22]. *P. falciparum* malaria transmission is considered hypoendemic in the region, since the incidence rates were estimated as 1.48 and 2.75 per 1,000 person-months in 6–59 months and 5–14 years old, respectively [23]. Our calculated incidence rate for children age 2–9 years old is 1.53 infections per 1,000 person-months (roughly equivalent to the infection rate 1–2% of tested population per year). Symptomatic cases clustered geographically and seasonally, with the highest case load in rainy season in May-October when 80% of cases were identified [23]. Previous observations in a large malaria survey of the population suggest that the rates of asymptomatic malaria may be 2–3 times higher than the symptomatic rates (unpublished data, BRAC). This paper describes a clinical field study to estimate the period prevalence of asymptomatic malaria infection among apparently healthy pregnant women, in comparison with non-pregnant women and adult men.

## Methods

### Study design, site and population

**Figure 1:**
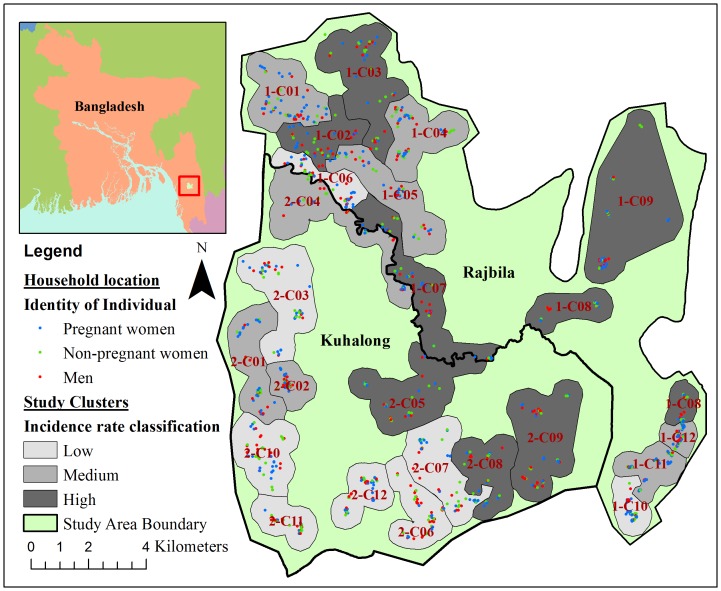
ArcGIS spatial analytical map for active surveillance of asymptomatic malaria. Red, green or blue colored dots represent the household locations of participating men, non-pregnant women, or pregnant women, respectively. Background dark, medium or light grey color represents the highest, medium or lowest eight incidence clusters, respectively.

This was a clinical longitudinal field surveillance study to assess if pregnancy is a risk factor for asymptomatic *P. falciparum* malaria. The study was conducted as part of a large passive and active malaria surveillance system established in the CHD of Bangladesh to study the epidemiology of malaria in the region, and to link demographic, clinical, and entomologic factors in the determination of important risk factors for malaria. The details of this study system and its methods of data collection have been published elsewhere [24]. Briefly, demographic and malaria surveillance was conducted in two demographically defined unions (Kuhalong and Rajbila) of the CHD, covering a population of approximately 24,000 individuals in 4,500 households over a defined area of 179 km^2^ (Figure 1). The region was divided into 24 study clusters in the two unions for programmatic purposes with each cluster containing approximately 1,000 individuals. Initial demographic and socioeconomic data were collected from October 2009 to February 2010 in Kuhalong and from April to August 2010 in Rajbila. Immigration, emigration, births and deaths of the study population were monitored and updated by trained surveillance workers every 3–4 months.

Passive and active malaria surveillance was initiated in Kuhalong union in October 2009 and Rajbila union in April 2010 by trained field workers. Data from active surveillance between May 2010 and January 2013 (total of 32 months of active surveillance) were included in this analysis. Active surveillance was conducted as cross-sectional, random screening of the population using a two-stage cluster sampling design, as previously described [24]. For the non-pregnant adult and children populations, 12 individuals per union were selected for active malaria screening per week stratified by age, with four individuals from each pre-specified age groups (<5 years, 5–14 years, ≥15 years). In each union, two of the 12 individuals were randomly selected and entered into a longitudinal surveillance cohort and tested for malaria every three months. Pregnant women were continually identified in the study area through the demographic surveillance system by field workers at the village level or through a pregnancy test for women selected into active surveillance. Selection of pregnant participants was independent of the study design for the passive and active surveillance in the non-pregnant populations. All pregnant women identified in the two study unions were invited to join the study. All consenting pregnant women were enrolled, placed in the longitudinal surveillance cohort, and tested for malaria every three months until delivery. After delivery, the pregnant women were counted as postpartum (within 6 months after delivery) and non-pregnant (after 6 months postpartum) and were continued to be tested for malaria every three months in the longitudinal surveillance cohort.

Individuals were tested for asymptomatic malaria infection by a rapid diagnostic test (RDT) using FalciVax test strip, which detects both *P. falciparum* and *P. vivax* malaria, and microscopic examination of thick and thin blood smears. The reported sensitivity and specificity of FalciVax RDT were relatively high (97.6% and 95.8% for *P. falciparum*, and 76.5% and 100% for *P. vivax*, respectively) in febrile patients who typically present with a medium-high parasite density [25]. However the detection ability of the RDT may be lower in asymptomatic parasitemic individuals where lower parasite density is expected [26]. Asymptomatic malaria infection was defined as a positive RDT or blood smear for *P. falciparum* in the absence of any clinical signs or symptoms suggestive of malaria, and only asymptomatic infections were included in this analysis. *P. vivax* infections were not included in this analysis because only one asymptomatic P. vivax infection was detected in the study population during the time period of the analysis. The presence of symptoms was subjectively investigated by general open-ended and specific questions, and body temperature was measured. Finger prick blood was collected for RDT and smear reading, and for retrospective PCR analysis. A positive infection was defined if either or both an RDT or blood smear was positive. A standard protocol for malaria microscopy quality management established at the International Center for Diarrhea Disease Research, Bangladesh (icddr,b) was followed, where a random sample of 100 positive and 100 negative samples were examined and independently validated by two microscopists not associated with the study. Positive RDT or blood smear, regardless of clinical symptoms, prompted a treatment with a standard three-day course of artemether-lumefantrine. Pregnant women positive for malaria during the first trimester were treated with quinine as per national treatment guidelines [13], although this event was rare. The first day of treatment was designated as day 0. Follow up of confirmed malaria took place on days 2, 7 and 28. At each visit, blood was collected and examined by smear microscopy to ensure the resolution of infection.

Detailed description of DNA extraction and real-time PCR (rt-PCR) analysis is available elsewhere [27]. Briefly, five 3 mm diameter punches, equivalent to 25 µl of whole blood, were removed from the filter papers. DNA was extracted with a commercial 96- well kit (Qiagen Mini Blood DNA extraction kit cat. no. 51106) and concentrated with a glycogen-acetate and acetate/ethanol precipitation and low-speed (3,000 g) centrifuge for 30 minutes. Multiplex rt-PCR amplified the 18S *P. falciparum* ribosomal gene with a Cy5 labeled probe. Samples were run in duplicate on manually loaded 96-well plates. A 40 cycle standard PCR protocol was run on a BioRad CFX96 PCR Detection System (BioRad, Hercules, CA, USA). Baseline relative fluorescence unit (RFU) values were readjusted within the Bio-Rad CFX manager software. The rt-PCR method demonstrated the highest sensitivity in the latent class analysis in febrile patients, and was able to detect 1–100 parasites per microliter. PCR testing was conducted for study samples collected between May 2010 and March 2012. Blood samples from non-endemic patients at Johns Hopkins University were used as negative control. Blood was also collected from the enrolled pregnant and non-pregnant women in the longitudinal surveillance, for the measurement of hemoglobin concentration using the hemocue system (Hemocue Hb 201, Hemocue America). Testing was performed in all women who were enrolled in the study from July 2011 to the end of the study.

Household locations for all individuals tested for malaria infection were mapped to show the spatial distribution of malaria tests in the study area. GPS coordinates for households were collected by local surveillance workers in March-April 2009, and mapped in ArcGIS (version 10.1; ESRI, Redlands USA). Using these GPS coordinates and passive malaria surveillance data, incidence clusters were defined based on the symptomatic *P. falciparum* incidence rates determined in a previous analysis [23]. These 24 study clusters were divided into three incidence categories containing eight clusters each labeled as “high incidence”, “medium incidence”, and “low incidence”.

### Statistical analysis

This study was designed to estimate the period prevalence of asymptomatic *P. falciparum* infection among pregnant women, non-pregnant women and adult men from May 2010 to January 2013, and investigated the relationship between asymptomatic malaria infection and the following risk factors over the same time period: pregnancy status, study union, the symptomatic malaria incidence of the study cluster, age, ethnicity, occupation, bed net use, and transmission season. Asymptomatic malaria infection was defined as a positive blood smear or RDT for *P. falciparum* malaria without any clinical signs or symptoms suggestive of malaria. The analysis was restricted to asymptomatic individuals 16–44 years old who were tested at least once by active surveillance between May 2010 and January 2013 and had complete lab data. Only day 0 tests were included in this analysis, and tests on follow-up days 2, 7 and 28 were excluded.

Age was stratified into two groups, those at or above the median age (25 years) and those below the median age. Anemia was defined as hemoglobin concentration less than 11.0 g/dL. Mild, moderate or severe anemia was defined as hemoglobin concentrations 10–10.9 g/dL, 7–9.9 g/dL, or less than 7 g/dL, respectively. Occupations of interest included housewives, agriculture, daily labor, jhum cultivation, and others (mostly students and unemployed). Jhum cultivation is a form of migrant agriculture in the hilly CHD region and has been implicated as a risk factor for symptomatic *P. falciparum* infection in this study area [23]. The temporality of asymptomatic infection was explored based on known seasonal transmission patterns: November to April as the low transmission season and May to October as the high transmission season. Other binary variables of interest in this analysis included study union (Rajbila or Kuhalong), ethnicity (non-tribal Bengali or tribal groups), and bed net use the previous night (yes or no). Study clusters were categorized as high, medium, and low incidence based on symptomatic *P. falciparum* incidence rates as described in the Methods section previously (Figure 1). Non-pregnant women were classified as postpartum if a test was taken within six months of the estimated date of delivery documented while she was pregnant (a proxy for the birth date) or non-postpartum if the test was taken later than six months after the estimated date of delivery or the women had never been pregnant.

The sociodemographic and temporal factors were compared between pregnant women, non-pregnant women and men ages 16–44 years using a Chi^2^ test. The period prevalence of asymptomatic malaria infection between pregnant women, non-pregnant women and men as well as within each of these risk categories were calculated and assessed for statistically significant differences using a Chi^2^ test. The period prevalence was calculated for both postpartum non-pregnant women and non-postpartum, non-pregnant women and compared using a Chi^2^ test.

The independent effect of pregnancy and other factors on asymptomatic *P. falciparum* infection was determined using multivariate logistic regression analysis. Dummy variables were created for demographic and temporal factors of interest and used to calculate the univariate and multivariate associations between these factors and asymptomatic malaria infection. Because very few asymptomatic *P. falciparum* cases occurred in the eight low incidence clusters, only data from the sixteen medium and high incidence clusters were used to maximize statistical power. The final multivariate logistic regression model was selected based using forward and backward stepwise regression (covariates were selected using a likelihood ratio test; p<0.1) after assessing the interactions between covariates. Models were assessed for fit using Hosmer-Lemeshow and Pearson's Chi^2^ goodness of fit tests and checked for collinearity by calculating variance inflation factors.

The effect of asymptomatic malaria infection and pregnancy status on blood hemoglobin level was assessed. The median and inter-quartile range of blood hemoglobin concentration (in g/dL) were calculated and presented as box plots for asymptomatic malaria cases and non-cases, pregnant women and non-pregnant women, and the interaction of the two. Statistical significance was determined using a non-parametric Kruskall-Wallis equality of populations test. The percent of each group that was moderately or severely anemic (<10 g/dL) was calculated for each group and assess for statistical significance using a Chi^2^ test. All statistical analyses were conducted in Stata (version 12.1; Statacorps, College Station, USA).

The overall period prevalence of asymptomatic malaria infection detected by PCR was calculated for pregnant women, non-pregnant women and men, and stratified into RDT- or smear-positive and PCR-positive infections that were negative by RDT or smear.

### Ethical considerations

A written informed consent or assent was obtained prior to enrollment from all adult or children (as defined as less than 16 years of age) participants, respectively. Permission from a guardian was also obtained for children. Additional consent was obtained at the time of enrollment into the active surveillance cohort. The study protocol and informed consent form were approved by the icddr,b Ethical Review Committee (PR#09021) and the Johns Hopkins Bloomberg School of Public Health Institutional Review Board (IRB#1965). The study was conducted in accordance with the principles of research ethics stated in the Declaration of Helsinki and the local and international regulatory guidelines.

## Results

### Study population characteristics

A total of 3,110 tests of malaria RDT and blood smear from individuals ages 16–44 years old were performed as part of active surveillance from May 2010 to January 2013. Among these, 909 tests were taken from 526 pregnant women, 1,753 tests from 911 non-pregnant women, and 448 tests from 316 men. Tests gathered from active surveillance were distributed evenly across the study area for men, pregnant women and non-pregnant women (Figure 1).

The demographics of these different groups are described in Table 1. Pregnant women were more likely to be from Rajbila, younger (61% of pregnant women were 16–24 years old compared to 44% and 31% of non-pregnant women and men, respectively), and more likely to be tested during the high transmission season (62% of pregnant women compared to 56% and 55% in non-pregnant women and men, respectively). Pregnant women, non-pregnant women and men also had significantly different occupation patterns and tended to live in different study clusters when study clusters were stratified by symptomatic *P. falciparum* incidence rates. There were no significant differences observed in ethnicity and bed net use among the three groups.

**Table 1:** Description of the study participants.

Demographic factors	Pregnant (%)	Non-pregnant (%)	Men (%)
***Union***			
Rajbila	538 (59.2)	872 (49.7)	219 (48.8)
Kuhalong	371 (40.8)	881 (50.3)	229 (51.1)
***Study Incidence cluster***			
Low Incidence	269 (29.6)	629 (35.9)	156 (34.8)
Medium Incidence	371 (40.8)	616 (35.1)	140 (31.3)
High Incidence	269 (29.6)	508 (29.0)	152 (33.9)
***Median age (25 years)***			
< median age (16–24)	554 (61.0)	769 (43.9)	139 (31.0)
≥ median age (25–44)	355 (39.1)	984 (56.1)	309 (69.0)
***Ethnicity***			
Bengali	175 (19.3)	331 (18.9)	85 (19.1)
Tribal	734 (80.8)	1,422 (81.1)	359 (80.1)
***Occupation***			
Housewife	399 (45.6)	669 (38.4)	1 (0.2)
Agriculture	175 (20.0)	425 (24.4)	162 (36.5)
Daily labor	76 (8.7)	159 (9.1)	83 (18.7)
Jhum cultivation	109 (12.4)	267 (15.3)	72 (16.2)
Other	117 (13.4)	221 (12.7)	126 (28.4)
***Bed net use***			
Yes	577 (89.2)	1,372 (91.1)	384 (88.5)
No	70 (10.8)	134 (8.9)	50 (11.5)
***Transmission season***			
High (May-August)	560 (61.6)	978 (55.8)	247 (55.1)
Low (Sept-April)	349 (38.4)	775 (44.2)	201 (44.9)
**Total**	909	1,753	448

### Risk factors for asymptomatic *P. falciparum* infection

The period prevalence of asymptomatic *P. falciparum* infection was assessed by calculating the percentage of tests that were positive for *P. falciparum* malaria infection by a RDT or blood smear microscopy from May 2010 to January 2013 (Table 2). There were a total of 34 asymptomatic infections (21 pregnant women, 9 non-pregnant women and 4 men), originated from 34 different participants. All 34 infections were RDT-positive and 27 of them were both RDT- and smear-positive. The median number (range) of tests per participant across study populations was 2 (1–4), 2 (1–7) and 1 (1–4) in pregnant women, non-pregnant adult women and adult men, respectively, and 43.9% (231/526), 44.7% (407/911), and 83.2% (263/316), of pregnant women, non-pregnant adult women and adult men were tested only once. 19 of 21 (90.5%), 3 of 9 (35%) and 3 of 4 (75%) of positive infections in the pregnant, non-pregnant and male population, respectively, were tested positive on the first testing. There were no recurrent infections after treatment or no repeated infection in the same individual. Pregnant women had the highest period prevalence of asymptomatic *P. falciparum* infection detected by RDT or smear (2.3%) compared to non-pregnant women (0.5%) and men (0.9%) (Chi^2^ test; p<0.001). Statistically significant risk factors for asymptomatic *P. falciparum* infection included residence in Rajbila union (1.6% compared to 0.5% in Kuhalong union), and younger age (1.7% in those 16–24 years old compared to 0.6% in those 25–44 years). Individuals living in study clusters of high symptomatic *P. falciparum* incidence had the highest period prevalence of asymptomatic infection (1.8%) compared to study clusters of medium (1.1%) and low incidence (0.5%). There were no significant differences in the period prevalence of different occupation groups, among those reporting bed net use the previous night compared to those who did not, and across tests taken in the high and low transmission seasons. Among non-pregnant women, there was no statistically significant difference between the period prevalence of asymptomatic malaria among those tests taken ≤6 months postpartum (4 cases among 800 tests; 0.5%) and those taken from women who have never been pregnant or >6 months postpartum (5 cases among 953 tests; 0.5%).

**Table 2:** Prevalence of asymptomatic malaria infection by demographics.

Demographic factors	Total	Malaria positive (%)	*p*-value
***Respondent tested***			
Pregnant women	909	21 (2.3)	<0.001
Non-pregnant women	1,753	9 (0.5)	
Men	448	4 (0.9)	
***Union***			
Rajbila	1,629	26 (1.6)	0.005
Kuhalong	1,481	8 (0.5)	
***Study incidence cluster***			
Low Incidence	1,054	5 (0.5)	0.015
Medium Incidence	1,127	12 (1.1)	
High Incidence	929	17 (1.8)	
***Median age (25 years)***			
< median age (16–24)	1,648	25 (1.7)	0.002
≥ median age (25–44)	1,462	9 (0.6)	
***Ethnicity***			
Bengali	591	2 (0.3)	0.050
Tribal	2,515	32 (1.3)	
***Occupation***			
Housewife	1,069	10 (0.9)	0.107
Agriculture	762	6 (0.8)	
Daily labor	318	1 (0.3)	
Jhum cultivation	448	8 (1.8)	
Other	464	9 (1.9)	
***Bed net use***			
Yes	2,333	18 (0.8)	0.490
No	254	3 (1.2)	
***Transmission season***			
High (May to August)	1,785	22 (1.2)	0.386
Low (September to April)	1,325	12 (0.9)	
Total	3,110	34 (1.1)	

Results of the PCR analysis were available for the first 2,362 (716 samples from 454 pregnant women, 1,242 from 690 non-pregnant women and 404 from 308 men) blood samples, collected from May 2010 to March 2012. Of these, a total of 67 samples (2.8%) were PCR-positive: 33 (4.6%), 23 (1.9%) and 11 (2.7%) in pregnant women, non-pregnant women, and men, respectively. Of these PCR-positive samples, 32 were also positive by RDT/smear. Therefore the PCR detected additional 35 infections that were negative by RDT or smear: 14 (2.0%), 14 (1.1%) and 7 (1.7%) samples in pregnant women, non-pregnant women, and men, respectively.

### The independent effect of pregnancy on asymptomatic *P. falciparum* infection

The unadjusted and adjusted odds of asymptomatic *P. falciparum* infection were calculated using univariate and multivariate logistic regression modeling (Table 3). Only data from study clusters of high or medium symptomatic *P. falciparum* incidence were used for this analysis (Figure 1). After using stepwise selections to generate a final multivariate logistic regression mode that accounts for the inter-relatedness of the final covariates, pregnant women had 5.4-fold higher odds of asymptomatic *P. falciparum* infection as compared to non-pregnant women (p = 0.001). Men had 2.5-fold higher odds of infection than non-pregnant women, however this relationship was not statistically significant (p = 0.214). Other factors selected into the final model included living in Rajbila union as compared to Kuhalong union (OR = 2.8; p = 0.072) and age, with the odds of infection decreasing by 15% per year of age (OR = 0.85; p = 0.001). The final model was assessed for goodness-of-fit and found to adequately fit the data (Pearson's Chi^2^ = 113.9, p = 0.995; Hosmer-Lemeshow Chi^2^ = 10.7, p = 0.221).

**Table 3:** Odds of asymptomatic infection in high & medium incidence clusters.

	Unadjusted		Adjusted				
Demographic factors	OR (95% CI)	*p*-value	OR (95% CI)			*p*-value	
***Respondent tested***							
Non-pregnant women	1.0		1.0				
Men	2.3 (0.6–9.8)	0.250	2.5 (0.6–10.6)			0.215	
Pregnant women	7.6 (2.8–20.2)	<0.001	5.4 (2.0–14.5)			0.001	
***Union***							
Kuhalong	1.0		1.0				
Rajbila	3.6 (1.2–10.2)	0.019	2.7 (0.9–7.8)			0.071	
***Age (continuous)***	0.83 (0.76–0.91)	<0.001	0.85 (0.77–0.93)			0.001	
***Ethnicity***							
Bengali	1.0		Not selected^b^				
Tribal	3.4 (0.5–25.4)	0.225					
***Occupation (N = 2021)*** a							
Agriculture	1.0						
Housewife	1.4 (0.4–4.5)	0.622		Not selected^b^			
Daily labor	0.5 (0.1–4.6)	0.551					
Jhum cultivation	2.2 (0.6–7.6)	0.211					
Other	3.1 (1.0–10.3)	0.059					
***Bed net use (N = 1,672)*** a							
No	1.0						
Yes	0.6 (0.2–2.2)	0.450		Not selected^b^			
***Transmission season***							
Low (Sept. to April)	1.0						
High (May to August)	1.3 (0.6–2.7)	0.555		Not selected^b^			

OR, odds ratio; 95% CI, 95% confidence interval;

a Missing data in demographic and bednet survey;

b Not selected into final multivariate model based on higher than 10% Type I error on LR test.

### The association between pregnancy and asymptomatic *P. falciparum* infection with blood hemoglobin

**Figure 2:**
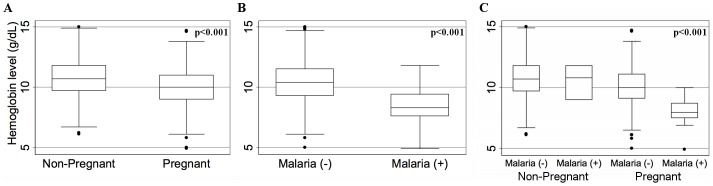
Box-plots of hemoglobin in pregnant and non-pregnant women with or without asymptomatic malaria. Box-plots represent the median and interquartile range of hemoglobin concentration. Panel **A** shows values in non-pregnant (n = 444) and pregnant (n = 495), **B**, malaria negative (n = 926) and malaria positive (n = 13), and **C**, the interaction of pregnancy and malaria indicating the lowest hemoglobin concentration in malaria-positive pregnant women (n = 10) compared with malaria-negative non-pregnant (n = 441) or pregnant (n = 485) women or malaria-positive non-pregnant women (n = 3). (Statistical significance by non-parametric Kruskal-Wallis equality of populations test)

Hemoglobin concentrations were available in 495 (54.5%) and 444 (25.3%) pregnant and non-pregnant women, respectively. The median and interquartile range of blood hemoglobin concentration was calculated and stratified by pregnancy status and asymptomatic malaria infection status (Figure 2). Overall, the median blood hemoglobin concentration was lower in pregnant than non-pregnant women (10.0 g/dL *vs*. 10.7 g/dL), and in women with than without asymptomatic falciparum infection (8.3 g/dL *vs*. 10.4 g/dL). The presence of both pregnancy and malaria appeared to interact and magnify the negative impact, as pregnant women with asymptomatic *P. falciparum* malaria had the lowest median blood hemoglobin (8.0 g/dL) compared to women with non-pregnant, malaria negative women (10.7 g/dL), non-pregnant, malaria positive women (10.8 g/dL), and pregnant, malaria-negative women (10.0 g/dL). A greater proportion of pregnant than non-pregnant women (51.5% *vs*. 33.3%, Chi^2^ test p<0.001) and malaria positive than malaria negative women (84.6% *vs*. 42.3%, Chi^2^ test p = 0.002) had moderate or severe anemia (hemoglobin less than 10 g/dL). Of 21 malaria positive pregnant women, 10 were tested for hemoglobin, and all were moderately to severely anemic. Of 9 malaria-positive non-pregnant women, 3 were tested for hemoglobin and 1 was moderately to severely anemic.

## Discussion

In this study of asymptomatic *P. falciparum* malaria in the Chittagong Hill Districts of Bangladesh, pregnant women had significantly higher period prevalence of asymptomatic *P. falciparum* infection compared to men and non-pregnant women over 32 months of active and longitudinal malaria surveillance. Using a real time PCR analysis we detected a similar ratio of malaria prevalence between pregnant women, non-pregnant women, and men, as compared to RDT or blood smear. RDT or smear detected about one-half of infections detected by rt-PCR. None of the submicroscopic infections detected by PCR progressed to clinical malaria.

This association held true in a multivariate logistic regression model controlling for maternal age and study union in the 16 study clusters with the highest malaria incidence. These results are consistent with other studies from high-transmission settings in sub-Saharan Africa [1] and among limited evidence from low-transmission settings [2], [28], [29] that show that pregnant women are at greater risk for *P. falciparum* infection.

This study shows two important findings. First, the temporal and spatial distributions of asymptomatic infections differ from that of symptomatic disease. These observations were made over a similar time period, excluding the possibility of changes in transmission over time potentially confounding the observed infections. We previously found that the incidence of symptomatic *P. falciparum* infection in all age groups was 6-fold higher in the high transmission season (May-October) than the low transmission season (November-April), and 18.3-fold higher in the high incidence cluster than in the low incidence clusters [23]. In this study, however, there was no significant difference in the period prevalence of asymptomatic *P. falciparum* infection between the two transmission seasons. While a subsequent analysis will investigate the epidemiology of asymptomatic infection in all age groups in this study area, our study results suggest that asymptomatic malaria infection is more evenly distributed across time and space than symptomatic infections. This observation will have a critical implication in the development of strategies for malaria control, particularly in the era of malaria elimination in regions such as Bangladesh where low transmission intensity justifies such an attempt. Further research is urgently needed to understand the nature and distribution of asymptomatic malaria infection serving as an important infected reservoir to continue malaria transmission.

Second, our observation in this study also suggested that *P. falciparum* infection and pregnancy synergistically contribute to maternal anemia in a hypoendemic malaria setting. Either pregnancy or malaria infection was associated with low hemoglobin concentration, and the women who were both pregnant and malaria positive had the lowest hemoglobin concentration. While hemoglobin values were available only in a portion of women, and the number of malaria-positive was too low to draw a definitive conclusion, the results are in agreement with evidence from high transmission settings [3], [7], [30]–[33], and support the limited evidence in low transmission settings [2], [34], [35], with one study in Ethiopia showing that malaria-positive pregnant women were more likely to be anemic in a low compared to a high transmission area [36].

While this study was grossly underpowered to detect the negative health consequences related to malaria during pregnancy, our findings echoed previous observations of detrimental effects of malaria in pregnant women and the fetus, and highlighted an urgent need to optimize the management of asymptomatic malaria infection in pregnant women residing in hypoendemic settings. While the risk-benefit balance may not favor of the use of IPTp, a mass preventive malaria treatment to minimize negative consequences of malaria in pregnant women widely used in regions of sub-Saharan Africa with moderate to high malaria transmission, screening of all pregnant women using RDT at prenatal visits may be effective. Further research is warranted to assess the effects of malaria detection and treatment targeting among this complex and vulnerable group of women.

The reported rate of the use of bed nets (ITN) was high in our study, but we did not systematically measure the effectiveness of, or the lack thereof, ITNs in our study populations. The use of ITNs is one of the strategies that has been shown in other studies to improve malaria control and childhood mortality and morbidity [37] and provide protection for pregnant women [38]. These findings should be interpreted with caution since methodological and statistical flaws have been reported [37] and some studies have reported paradoxical findings that increased ITN use is associated with increased malaria incidence in the face of ITN distribution [39]. Data on ITN effectiveness are sparse in low transmission countries such as Bangladesh, and are critically needed to examine the impact of ITN on asymptomatic infection as well as anemia in pregnant women of such settings.

There are a number of important limitations to this study. First, we performed repeated testing in the same individual and the effect of non-independent measurement may have systematically biased our results. However, for this analysis, we assume that data points are independent since all infections originated from independent participants and the majority of positive infections were positive on the first testing. It is, therefore, unlikely that our study finding can be explained by a systematic error of repeated testing, or by the disproportionate testing frequency in one study population over the other. Similarly, this assumption of independence among repeated testing in the same individual may not be true since previous malaria infection may provide some immunity to subsequent infections. However, the likelihood that this assumption biased the results in this hypoendemic setting is low. Second, the study is underpowered with only 34 asymptomatic *P. falciparum* infections, including only two infections in non-tribal Bengalis. These low numbers could not accommodate a multivariate model to assess potentially important factors risking over-stratification of the data and leading the large confidence intervals. Therefore, important confounders and effect modifiers of the effect of pregnancy on malaria infection may be missing from the model. Despite this limitation, this study was still powered enough to detect a clinically relevant 2–4 fold increase in the period prevalence of *P. falciparum* infection in pregnant women compared to non-pregnant women, adjusted for age and study location. Third, there is potential discrepancy between the estimated date of delivery and actual date of pregnancy termination, raising a risk of incorrect assignment of some women who had miscarriage or premature delivery. However we were able to confirm the date of pregnancy termination in 28 of 30 women, using information on the delivery outcome in the demographic surveillance system, and their status of pregnancy at the time of the detection of asymptomatic malaria infection. Moreover we were able to measure hemoglobin only in a portion of study women, and did not examine the association between asymptomatic malaria risk and gestational age of pregnancy, gravidity or parity, or pregnancy outcomes, for which a larger stratified study will be needed. Similarly, we did not address the impact of pregnancy on *P. vivax* infection, an important known cause of pregnancy anemia and maternal and infant morbidity [35], or symptomatic malaria disease. Further work is needed to identify the relationship between *P. vivax* infection and pregnancy status in Bangladesh, especially in areas where *P. vivax* is more common.

## Conclusion

In summary, this study shows that the risk of asymptomatic *P. falciparum* infection is significantly higher in pregnant women than in non-pregnant adult women or men in the Chittagong Hills Districts of Bangladesh, and suggests that the presence of *P. falciparum* infection in pregnant women may heighten the risk of anemia. These results underscore the need to further evaluate ways to optimize management of malaria infection in pregnant women living in areas where malaria transmission intensity is low. Management of asymptomatic infections at a population level, particularly in a low transmission setting, is complex owing to the risk-benefit ratio as well as to its social acceptance. Pregnant women present a different and important argument due to the known risk of malaria, regardless of symptomatic illness, to the mother and fetus. While argument for mass treatment such as IPTp widely used in high-transmission settings in sub-Saharan Africa may not be favorable in areas of low malaria transmission intensity such as Bangladesh, active screening and prompt treatment, with or without clinical symptoms, should be considered for high-risk populations. Our study also shows that asymptomatic *P. falciparum* infections, the most critical and problematic parasite population in breaking the transmission cycle, are evenly distributed throughout time and space, differing from the observed pattern of symptomatic infections which tend to cluster geographically and with time. These findings have critical implications for malaria control or elimination strategies. Further research is warranted to understand the distribution of asymptomatic infected reservoir, and to assess ways to eradicate it at the population level.
